# Supplementary material for: Content validation of an activity-based therapy tracking tool in a community setting for people living with spinal cord injury or disease using cognitive debriefing interviews
Source: PLoS One. 2024 Dec 30;19(12):e0315404. doi: 10.1371/journal.pone.0315404 (PMC11684641; doi:10.1371/journal.pone.0315404)
Supplement: S1 Text — (DOCX) [file pone.0315404.s001.docx]

**Activity-Based Therapy (ABT) Tracking Tool**

Thank you for agreeing to provide feedback on the content of the ABT Tracking Tool. This is an important step in the creation of the tracking tool, which will eventually exist in electronic form (i.e. an app). This version of the ABT Tracking Tool was developed from findings from the following: 1) a scoping review; 2) twelve focus group meetings with stakeholders; and 3) a Delphi survey.

When completing an ABT session, please consider whether the content of this tool accurately capture the different exercises performed. Some ABT exercises may not be relevant to your ABT program or session. Please only complete the sections for exercises that you completed. Each page is dedicated to a different ABT exercise.

Treadmill Training page 2

Overground Walking page 3

Muscle Strengthening page 4

Ergometer Training page 5

Load-bearing Exercise: Standing page 6

Load-bearing Exercise: 4 point page 7

Load-bearing Exercise: High Kneel page 8

Load-bearing Exercise: Crawling page 9

Transfer Training page 10

Balance Training page 11

Other Task-specific Movement page 12


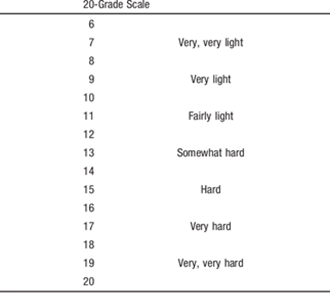


**Treadmill Walking**

*This ABT activity refers to any walking practice that is completed on a treadmill.*

Total duration of activity: _______ minutes

Duration of walking bout(s) and rest (s)

| Bout/Rest Number | Walk (minutes) | Rest (minutes) |
| --- | --- | --- |
| 1 |  |  |
| 2 |  |  |
| 3 |  |  |
| 4 |  |  |
| 5 |  |  |

Walking speed (range or max): ________
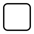
 meters/second
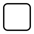
 miles/hour


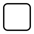
 Body weight support: _____ (% of body weight supported)

Total distance walked: ________
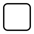
 metres
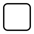
 miles

--------------------------------------------------------------------------------------------------------

Number of people assisting: _______


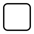
 Left leg
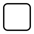
 Right leg
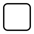
 Pelvis/trunk
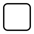
 Arms

Overall level of assistance:


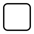
 Minimal (i.e. participant provides 75% or more of the effort)


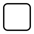
 Moderate (i.e. participant provides 50-74% of the effort)


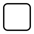
 Maximal (i.e. participant provides 25-49% of the effort)


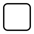
 Total assist (i.e. participant provides less than 25% of the effort)

Upper limb support:
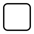
 Side rail
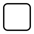
 Forward rail Rail height: _____
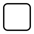
 meters
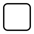
 inches


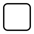
 Braces: _______ (e.g. AFO, KAFO)

--------------------------------------------------------------------------------------------------------

Additional levels of difficulty:


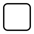
 Incline
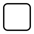
 Decline
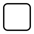
 Backward walking


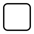
 Sideway walking
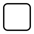
 Obstacle walking
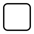
 Added resistance (e.g. weights, bands)

Details: _______________________________________________________________________

______________________________________________________________________________

--------------------------------------------------------------------------------------------------------


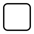
With robotic device (e.g. Lokomat)

Guide force (N): _______ Resistive force (N): _______

--------------------------------------------------------------------------------------------------------

Measures of exertion:


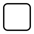
 Borg Rating of Perceived Exertion: ______


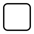
 Heart rate maximum: ________
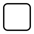
 Blood Pressure maximum: ________

--------------------------------------------------------------------------------------------------------


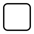
 With neuromuscular electrical stimulation

Muscles/peripheral nerves targeted: __________________________________

Phase of gait cycle targeted:
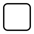
 stance
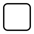
 swing

Waveform:
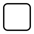
 symmetric
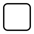
 asymmetric Frequency (Hz or pps): ________

Pulse duration (µs): _______ Intensity (mA): ________

**Overground Walking**

*This ABT activity refers to any walking practice that is completed over the ground.*

Total duration of activity: _______ minutes

Duration of walking bout(s) and rest (s)

| Bout/Rest Number | Walk (minutes) | Rest (minutes) |
| --- | --- | --- |
| 1 |  |  |
| 2 |  |  |
| 3 |  |  |
| 4 |  |  |
| 5 |  |  |

Walking speed (range or max): ________
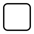
 meters/second
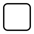
 miles/hour


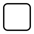
 Body weight support: _____ (% of body weight supported)

Total distance walked: ________
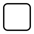
 metres
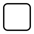
 miles

--------------------------------------------------------------------------------------------------------

Number of people assisting: _______


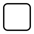
 Left leg
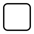
 Right leg
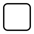
 Pelvis/trunk
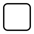
 Arms

Overall level of assistance:


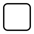
 Minimal (i.e. participant provides 75% or more of the effort)


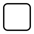
 Moderate (i.e. participant provides 50-74% of the effort)


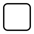
 Maximal (i.e. participant provides 25-49% of the effort)


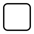
 Total assist (i.e. participant provides less than 25% of the effort)


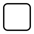
 Braces: _______ (e.g. AFO, KAFO)


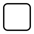
 Walking aid: _______ (e.g. cane, 2-wheeled walker, 4-wheeled walker, forearm crutches)

--------------------------------------------------------------------------------------------------------

Additional levels of difficulty:


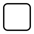
 Incline
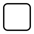
 Decline
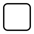
 Backward walking


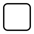
 Sideway walking
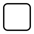
 Obstacle walking
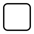
 Added resistance (e.g. weights, bands)


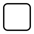
 Challenging walking surface: ________________

Details: _______________________________________________________________________

______________________________________________________________________________

--------------------------------------------------------------------------------------------------------

Measures of exertion:


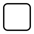
 Borg Rating of Perceived Exertion: ______


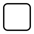
 Heart rate maximum: ________
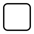
 Blood Pressure maximum: ________

--------------------------------------------------------------------------------------------------------


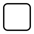
 With neuromuscular electrical stimulation

Muscles/peripheral nerves targeted: __________________________________

Phase of gait cycle targeted:
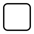
 stance
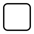
 swing

Waveform:
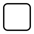
 symmetric
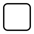
 asymmetric Frequency (Hz or pps): ________

Pulse duration (µs): _______ Intensity (mA): ________

**Muscle Strengthening**

*This ABT activity is also called resistance training. It includes active assisted exercises and may involve weight machines or free weights.*

Total duration of activity: _______ minutes

Strengthening exercise: ________________________

Muscle(s) targeted: _____________________________


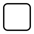
 With external load: _____
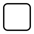
 pounds
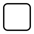
 kilograms
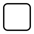
 Newtons

Sets, repetitions, and rest(s)

| Set Number | Number of Repetitions | Rest (minutes) |
| --- | --- | --- |
| 1 |  |  |
| 2 |  |  |
| 3 |  |  |
| 4 |  |  |
| 5 |  |  |

--------------------------------------------------------------------------------------------------------

Number of people assisting: _______

Overall level of assistance:


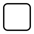
 Minimal (i.e. participant provides 75% or more of the effort)


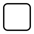
 Moderate (i.e. participant provides 50-74% of the effort)


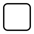
 Maximal (i.e. participant provides 25-49% of the effort)


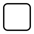
 Total assist (i.e. participant provides less than 25% of the effort)

--------------------------------------------------------------------------------------------------------

Measures of exertion:


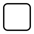
 Borg Rating of Perceived Exertion: ______


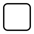
 Heart rate maximum: ________
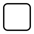
 Blood Pressure maximum: ________

--------------------------------------------------------------------------------------------------------


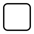
 With neuromuscular electrical stimulation

Muscles/peripheral nerves targeted: __________________________________

Waveform:
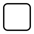
 symmetric
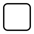
 asymmetric

Frequency (Hz or pps): ________

Pulse duration (µs): _______ Intensity (mA): ________

On time (s): ________ Off time (s): ________

**Ergometer Training**

*This ABT activity involves cycling movements of the arms and/or legs. Ergometer training includes arm crank, hand cycle, leg cycle, stationary bike, spin bike, arm and leg ergometer, and recumbent tricycle.*

Total duration of activity: _______ minutes


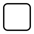
 Leg ergometry
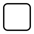
 Arm ergometry
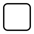
 Arm and leg ergometry


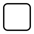
 Recumbent


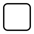
 With back support (e.g. wheelchair)

Duration of cycling bout(s) and rest (s)

| Bout/Rest Number | Cycle (minutes) | Rest (minutes) |
| --- | --- | --- |
| 1 |  |  |
| 2 |  |  |
| 3 |  |  |
| 4 |  |  |
| 5 |  |  |

Cadence (range or max): ________ revolutions/minute


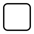
 With resistance: details ______________________________________

Power output: ________
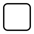
 Watts
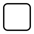
 kilogram meters/minute

--------------------------------------------------------------------------------------------------------

Number of people assisting: _______


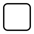
 Left leg
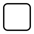
 Right leg
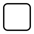
 Pelvis/trunk
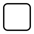
 Arms

Overall level of assistance:


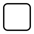
 Minimal (i.e. participant provides 75% or more of the effort)


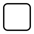
 Moderate (i.e. participant provides 50-74% of the effort)


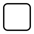
 Maximal (i.e. participant provides 25-49% of the effort)


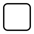
 Total assist (i.e. participant provides less than 25% of the effort)

--------------------------------------------------------------------------------------------------------

Measures of exertion:


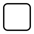
 Borg Rating of Perceived Exertion: ______


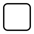
 Heart rate maximum: ________
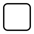
 Blood Pressure maximum: ________

--------------------------------------------------------------------------------------------------------


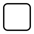
 With neuromuscular electrical stimulation

Muscles/peripheral nerves targeted: __________________________________

Phase of pedal cycle targeted:
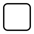
 power (extension)
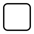
 recovery (flexion)

Waveform: symmetric asymmetric

Frequency (Hz or pps): ________

Pulse duration (µs): _______ Intensity (mA): ________

**Load-bearing Exercise**

*This ABT activity involves supporting your own body weight. It includes standing, high kneeling, crawling and holding a quadruped (i.e., 4-point) position.*

**Load-bearing Exercise: Standing**

Total duration of activity: _______ minutes

Duration of bout(s) and rest (s)

| Bout/Rest Number | Load-bearing (minutes) | Rest (minutes) |
| --- | --- | --- |
| 1 |  |  |
| 2 |  |  |
| 3 |  |  |
| 4 |  |  |
| 5 |  |  |

Body weight support: _____ (% of body weight supported)

With standing frame With tilt table

--------------------------------------------------------------------------------------------------------

Number of people assisting: _______

Left leg Right leg Pelvis/trunk Arms

Overall level of assistance:

Minimal (i.e. participant provides 75% or more of the effort)

Moderate (i.e. participant provides 50-74% of the effort)

Maximal (i.e. participant provides 25-49% of the effort)

Total assist (i.e. participant provides less than 25% of the effort)

With upper extremity support:

Parallel bars Walker Forearm crutches Canes Forward rail

Braces: _______ (e.g. AFO, KAFO)

--------------------------------------------------------------------------------------------------------

Measures of exertion:

Borg Rating of Perceived Exertion: ______

Heart rate maximum: ________ Blood Pressure maximum: ________ --------------------------------------------------------------------------------------------------------

With neuromuscular electrical stimulation

Muscles/peripheral nerves targeted: __________________________________

Waveform: symmetric asymmetric

Frequency (Hz or pps): ________

Pulse duration (µs): _______ Intensity (mA): ________

On time (s): ________ Off time (s): ________

**Load-bearing Exercise: 4-point**

Total duration of activity: _______ minutes

Duration of bout(s) and rest (s)

| Bout/Rest Number | Load-bearing (minutes) | Rest (minutes) |
| --- | --- | --- |
| 1 |  |  |
| 2 |  |  |
| 3 |  |  |
| 4 |  |  |
| 5 |  |  |

Body weight support: _____ (% of body weight supported)

--------------------------------------------------------------------------------------------------------

Number of people assisting: _______

Left leg Right leg Pelvis/trunk Arms

Overall level of assistance:

Minimal (i.e. participant provides 75% or more of the effort)

Moderate (i.e. participant provides 50-74% of the effort)

Maximal (i.e. participant provides 25-49% of the effort)

Total assist (i.e. participant provides less than 25% of the effort)

--------------------------------------------------------------------------------------------------------

Measures of exertion:

Borg Rating of Perceived Exertion: ______

Heart rate maximum: ________ Blood Pressure maximum: ________ --------------------------------------------------------------------------------------------------------

With neuromuscular electrical stimulation

Muscles/peripheral nerves targeted: __________________________________

Waveform: symmetric asymmetric

Frequency (Hz or pps): ________

Pulse duration (µs): _______ Intensity (mA): ________

On time (s): ________ Off time (s): ________

**Load-bearing Exercise: High Kneel**

Total duration of activity: _______ minutes

Duration of bout(s) and rest (s)

| Bout/Rest Number | Load-bearing (minutes) | Rest (minutes) |
| --- | --- | --- |
| 1 |  |  |
| 2 |  |  |
| 3 |  |  |
| 4 |  |  |
| 5 |  |  |

Body weight support: _____ (% of body weight supported)

--------------------------------------------------------------------------------------------------------

Number of people assisting: _______

Left leg Right leg Pelvis/trunk Arms

Overall level of assistance:

Minimal (i.e. participant provides 75% or more of the effort)

Moderate (i.e. participant provides 50-74% of the effort)

Maximal (i.e. participant provides 25-49% of the effort)

Total assist (i.e. participant provides less than 25% of the effort)

With upper extremity support:

Side rail/support Forward rail/support

--------------------------------------------------------------------------------------------------------

Measures of exertion:

Borg Rating of Perceived Exertion: ______

Heart rate maximum: ________ Blood Pressure maximum: ________ --------------------------------------------------------------------------------------------------------

With neuromuscular electrical stimulation

Muscles/peripheral nerves targeted: __________________________________

Waveform: symmetric asymmetric

Frequency (Hz or pps): ________

Pulse duration (µs): _______ Intensity (mA): ________

On time (s): ________ Off time (s): ________

**Load-bearing Exercise: Crawling**

Total duration of activity: _______ minutes

Hands and knees crawl Army crawl Other type of crawl: ___________________

Duration of bout(s) and rest (s)

| Bout/Rest Number | Load-bearing (minutes) | Rest (minutes) |
| --- | --- | --- |
| 1 |  |  |
| 2 |  |  |
| 3 |  |  |
| 4 |  |  |
| 5 |  |  |

Crawling speed (range or max): ________ meters/second miles/hour

Body weight support: _____ (% of body weight supported)

Total distance crawled: ________ metres feet

--------------------------------------------------------------------------------------------------------

Number of people assisting: _______

Left leg Right leg Pelvis/trunk Arms

Overall level of assistance:

Minimal (i.e. participant provides 75% or more of the effort)

Moderate (i.e. participant provides 50-74% of the effort)

Maximal (i.e. participant provides 25-49% of the effort)

Total assist (i.e. participant provides less than 25% of the effort)

--------------------------------------------------------------------------------------------------------

Additional levels of difficulty:

Incline Decline Backward crawling

Side crawling Obstacle crawling Added resistance (e.g. weights, bands)

Challenging crawling surface: ________________

Details: _______________________________________________________________________

______________________________________________________________________________

--------------------------------------------------------------------------------------------------------

Measures of exertion:

Borg Rating of Perceived Exertion: ______

Heart rate maximum: ________ Blood Pressure maximum: ________ --------------------------------------------------------------------------------------------------------

With neuromuscular electrical stimulation

Muscles/peripheral nerves targeted: __________________________________

Phase of crawling: stance (limb extension) swing (limb flexion)

Waveform: symmetric asymmetric

Frequency (Hz or pps): ________

Pulse duration (µs): _______ Intensity (mA): ________

**Transfer Training**

*This ABT activity involves moving from one surface to another, such as from a wheelchair to a bed, or moving from one position to another, such as sitting to standing.*

Total duration of activity: _______ minutes

Starting position/surface of transfer: ____________________

Starting seat height: ____ cm inches

Ending position/surface of transfer: ____________________

Sets, repetitions, and rest(s)

| Set Number | Number of Repetitions | Rest (minutes) |
| --- | --- | --- |
| 1 |  |  |
| 2 |  |  |
| 3 |  |  |
| 4 |  |  |
| 5 |  |  |

--------------------------------------------------------------------------------------------------------

Number of people assisting: _______

Left leg Right leg Pelvis/trunk Arms

Overall level of assistance:

Minimal (i.e. participant provides 75% or more of the effort)

Moderate (i.e. participant provides 50-74% of the effort)

Maximal (i.e. participant provides 25-49% of the effort)

Total assist (i.e. participant provides less than 25% of the effort)

Braces: _______ (e.g. AFO, KAFO)

Assistive device: _______ (e.g. walker, forearm crutches, sit-to-stand trainer, transfer board)

--------------------------------------------------------------------------------------------------------

Measures of exertion:

Borg Rating of Perceived Exertion: ______

Heart rate maximum: ________ Blood Pressure maximum: ________ --------------------------------------------------------------------------------------------------------

With neuromuscular electrical stimulation

Muscles/peripheral nerves targeted: __________________________________

Waveform: symmetric asymmetric

Frequency (Hz or pps): ________

Pulse duration (µs): _______ Intensity (mA): ________

On time (s): ________ Off time (s): ________

**Balance Training**

*This ABT activity includes exercises that challenge stability in seated or upright positions.*

Total duration of activity: _______ minutes

Balance task: ________________________________________________

Position: sitting standing other: _______________________

Support surface: hard soft (e.g., foam, mat) uneven other: _______________

With visual input reduced

Duration of exercise bout(s) and rest (s)

| Bout/Rest Number | Bout (minutes) | Rest (minutes) |
| --- | --- | --- |
| 1 |  |  |
| 2 |  |  |
| 3 |  |  |
| 4 |  |  |
| 5 |  |  |

Body weight support: _____ (% of body weight supported)

--------------------------------------------------------------------------------------------------------

Number of people assisting: _______

Left leg Right leg Pelvis/trunk Arms

Overall level of assistance:

Minimal (i.e. participant provides 75% or more of the effort)

Moderate (i.e. participant provides 50-74% of the effort)

Maximal (i.e. participant provides 25-49% of the effort)

Total assist (i.e. participant provides less than 25% of the effort)

Braces: _______ (e.g. AFO, KAFO)

Assistive device: _______ (e.g. cane, walker, forearm crutches, parallel bars) --------------------------------------------------------------------------------------------------------

Measures of exertion:

Borg Rating of Perceived Exertion: ______

Heart rate maximum: ________ Blood Pressure maximum: ________ --------------------------------------------------------------------------------------------------------

With neuromuscular electrical stimulation

Muscles/peripheral nerves targeted: __________________________________

Waveform: symmetric asymmetric

Frequency (Hz or pps): ________

Pulse duration (µs): _______ Intensity (mA): ________

On time (s): ________ Off time (s): ________

**Other Task-specific Movement**

*This ABT activity refers to discrete, goal-directed movements of the arms or legs, such as reaching and grasping or kicking a ball.*

Total duration of activity: _______ minutes

Task-specific movement: ________________________

Starting position: _________________________

Muscle(s) targeted: _____________________________

With external load: _____ pounds kilograms Newtons

Sets, repetitions, and rest(s)

| Set Number | Number of Repetitions | Rest (minutes) |
| --- | --- | --- |
| 1 |  |  |
| 2 |  |  |
| 3 |  |  |
| 4 |  |  |
| 5 |  |  |

--------------------------------------------------------------------------------------------------------

Number of people assisting: _______

Left leg Right leg Pelvis/trunk Arms

Overall level of assistance:

Minimal (i.e. participant provides 75% or more of the effort)

Moderate (i.e. participant provides 50-74% of the effort)

Maximal (i.e. participant provides 25-49% of the effort)

Total assist (i.e. participant provides less than 25% of the effort)

--------------------------------------------------------------------------------------------------------

Measures of exertion:

Borg Rating of Perceived Exertion: ______

Heart rate maximum: ________ Blood Pressure maximum: ________ --------------------------------------------------------------------------------------------------------

With neuromuscular electrical stimulation

Muscles/peripheral nerves targeted: __________________________________

Waveform: symmetric asymmetric

Frequency (Hz or pps): ________

Pulse duration (µs): _______ Intensity (mA): ________

On time (s): ________ Off time (s): ________
