# Supplementary material for: Content validation of an activity-based therapy tracking tool in a community setting for people living with spinal cord injury or disease using cognitive debriefing interviews
Source: PLoS One. 2024 Dec 30;19(12):e0315404. doi: 10.1371/journal.pone.0315404 (PMC11684641; doi:10.1371/journal.pone.0315404)
Supplement: S2 Text — (DOCX) [file pone.0315404.s002.docx]

**Supplement 2. Interview guide**

**Interview Guide**

Participant Code: _______

Date:

Interviewer Initials:

Thank you for agreeing to participate in this study. We will be audio recording this meeting. If you could please try to refrain from using names or any other identifying information during the meeting, that would be appreciated. I’d also like to remind you that you can choose to not answer any of the questions that I will be asking in this meeting. I want to start by giving you a little background information about this project. We are conducting interviews with people living with spinal cord injury/disease (SCI/D) and clinicians who work with people with SCI/D to evaluate the content validity of an activity-based therapy (ABT) tracking tool. We would like to get your feedback on the content included in the tool, its usability, and your preferred delivery method. We’d also like to know of potential implementation barriers, if any. Before we begin the interview and start the recording, we first need to obtain your consent to participate in this study and then we also need to complete a short demographic and injury-related info form.

So first I would like to confirm that you still agree to participate in this study? [IF YES: “I will begin recording now”.] [IF NO: Interview will not be conducted.]

Do you have the ABT tracking tool with you? If you have it, please keep it with you for the interview.

I will begin recording the interview now:

You have been asked to participate in this study to reflect on your use of the ABT tracking tool.

1. To begin, can you describe how you used the ABT Tracking Tool, including how many times you used it?
   1. When did you use it? <Probe: *during or after the session*>
   2. Are you tracking right after the session, later in the evening or week?
      1. Did you find it difficult to remember the details of the information that you wanted to record? Especially if it was later that evening or week?
   3. What did you do to remind yourself to track the sessions?
   4. For the sessions that you did, did you do the same activities in each session or were they different?
   5. How long was each session that you did (i.e. duration)?
2. Do you think the activities outlined in the current ABT Tracking Tool represent all the possible activities that might be included in your/your clients’ ABT program
   1. If no, what activities are missing?
   2. If yes, was there anything that you think may have been missed? Even if you did not do this exercise, but maybe it is an exercise you have seen other people do?
   3. Do you think the ABT Tracking Tool is appropriate for people with all levels and severities of injury, where there are varying levels of function?

For this next question we are going to go through the tool.

[Repeat this section for each exercise]

1. What were the activities or exercises listed in the tool that you participated in during those 3 sessions? <Probe: *such as strengthening, load-bearing exercises, etc.*>
   1. So, I will go through each exercise listed in a similar format of questions. So starting with ….. Please find that page first before I begin.
      1. Do you feel that the characteristics/parameters listed on the tool for this ABT activity captures all the information you want to track? (i.e. are they appropriate and do they adequately capture the breadth of the training session?)
         1. Such as the number of repetitions, level of assistance, NMES parameters
      2. Was there anything that you feel is missed or could be added?
         1. How would you add this to the tool?
      3. Were the units of measure included appropriate for what you would be using? (lb & kg, m/s)
         1. What would you put instead?
      4. Was there anything you felt was unnecessary and can be removed?

So, I just wanted to know, with respect to the remaining exercises that were not done during the sessions, have you ever done any of them before? I was just wondering if you had a chance to look over them on the tracking tool and if you could comment on any of them (ask them if it would be okay if they take a quick look at it).

Do you feel that the characteristics/parameters listed captures all the information you want to track? Anything missed and could be added? Were the units of measure appropriate? Was there anything that you feel could be removed

1. What was easy about using the ABT Tracking Tool, if anything?
   1. <Probe: *Did you find it straightforward and detailed?*>
2. What was challenging about using the ABT Tracking Tool, if anything?
   1. <Probe: *Did you have difficulty figuring out where to record any of the activities you participated in?*>
3. How could the ABT Tracking Tool be improved? We want this tool to be available for people with SCI as well as the clinicians, so if there is anything you can think of that would make it easier for you to use the tool, we would love to hear your feedback.
   1. Did they like/dislike the general format/layout of the tool? (i.e. did you like that each exercise had its own page/measurements?)
4. How would you prefer to track your sessions? <Probe: *For example, on paper, app, another platform?*>
5. Do you anticipate any challenges in the implementation of this ABT tracking tool? (i.e. getting it out there for people with SCI or clinicians to be able to use?)
   1. If yes, what do you anticipate are those barriers or challenges?
   2. Do you have suggestions for addressing these barriers?
6. How do you propose us being able to get the word out and get people interested in taking up and using the tool? Are there specific social media platforms that would gain more exposure to the ABT tool?
7. If we are thinking about developing this into an app and if you were to use a tool like this, would you want to be able to create a user profile that is stored in an app? <Further elaboration: this way you would have your specificities and progress stored. You could keep track of progress and which locations they occurred at (such as ABT at inpatient/outpatient vs community vs home. Also, level/severity of injury and if that changes over time)>
8. What information do you think should be stored in this tool? <Probe: *What do you think would be helpful for you or the (clinician/client)? For example, demographic information such as age, sex, level of injury, time post-injury, setting, date of session, length of session*>
9. Is there anything regarding the functions and features of the app that we can do to make it easier to use? (Example: such as being able to pre-select parameters that are relevant to client only?)
10. Is there anything else you’d like to add about your experience using the ABT tracking tool?

We want to thank you for your time today. If you would like to read a transcript of the interview, it can be mailed to you. You can review it and then let us know if you have any requested additions, deletions, or revisions to make. Thank you. End.
