# Supplementary material for: Content validation of an activity-based therapy tracking tool in a community setting for people living with spinal cord injury or disease using cognitive debriefing interviews
Source: PLoS One. 2024 Dec 30;19(12):e0315404. doi: 10.1371/journal.pone.0315404 (PMC11684641; doi:10.1371/journal.pone.0315404)
Supplement: S1 Table — (DOCX) [file pone.0315404.s003.docx]

**Supplement 3. Suggested modifications to the ABT Tracking Tool**

| **Type of ABT** | **Suggested Revision** | **Example Quote** |
| --- | --- | --- |
| All | - Add a comment box | “A small comment section could be nice to put anything that isn’t already in the tool.” CL07 |
|  | - Add ‘Goal of exercise’ | “One thing that I would maybe add in is like the goal of the exercise. So, for example, I was doing a Bosu crunch with the client and you've got the strengthening exercises and you’ve got the muscles targeted, but what is the goal? Because someone could be doing that exercise for abdominal strengthening, but someone could also be doing it for like a dynamic balance.” CL03 |
|  | - Add ‘Technology and/or equipment used’ | “…a little category for type of equipment.” CL10 |
|  | - Include ‘Measures of exertion’ for each bout | “I really like seeing the exertion throughout the exercise [each rep], just to give you a little bit of a subjective measure of fatigue…if there was any one change I would love, it’s just [to] have that measure of exertion with the exercise so you can track fatigue…‘cause I really like having the measure of exertion, I would just love to be able to track it over bouts of exercise.” CL08 |
|  | - Expand parameter ‘Overall level of assistance’ to include ‘Full assistance, Full independence, Spotting and Verbal/Tactile cues’ | “I think relevant to all the other sections of the tool is just adding like 100% independent for overall level of assistance.” CL08 |
|  | - Add ‘Talk test’ to ‘Measures of exertion’ | “Breath rate is something that I consider. Like the Talk Test. So, whether they can sing, talk, gasp I think is the final one? That’s a good exertion level test to find out how hard someone's working.” CL05 |
|  | - Add check box for ‘Duration’ if they reached fatigue limit | “Unless you just want to say until fatigue…‘cause sometimes we don’t go, ‘do it this long and then rest and then do it this long and then rest for this long’, it’ll literally be til they can’t do it and then we’ll rest for a timed amount and then we’ll go again until they hit fatigue…Other cases, it’s just going to be a timed thing. You’re going to go 15 minutes and then you’re going to rest and they never reach fatigue so you don’t know when you’re making that progress.” CL09 |
|  | - Add parameter ‘Assistance from equipment’ (i.e., safety set-up, support, assist) and open space to indicate type of set-up and level of assistance provided by the equipment | “Say I have a client doing a sit-up off a Bosu and they can't do it and they’re in a short-seated position sitting on the edge of the bed, Bosu behind them. They’re doing a sit-up and I give them a six-pound dowel, something that's gonna assist them through the motion, how would I have tracked that on this page?...If I'm trying to get them to do an arm exercise, let's say they're trying to regain any sort of muscle fibers in the deltoid muscle and they can't exactly lift their arm, but if I give them a weight that's attached to a pully system…the weight counteracts the weight of their arm and helps them lift with the weight, if that makes sense. So it’s an active assisted exercise without the trainer.“ CL04 |
|  | - Add ‘Level of assistance’ for each body part | They were able to produce the rotations independently. The only assistance that I was providing was knee abduction. So there knees typically would collide inwards in adduction and possibly hit the bike. So, we had two trainers that were assisting with the abduction, but not actually for the extension or flexion phases of the cyclical motion…that's the only tricky part...I didn't feel like it was missing anything other than how I record that setup…I gave them slight abduction assistance and maximally I was maximally helping him in abduction, but not with the actual rotational motion that he was providing.” CL04 |
|  | - Add ‘Other’ as a new page | “If you provided an empty page in the same format [where] we were to add in stuff that we feel didn’t target these specific pages that you made already, [it] might have been beneficial…because you then get an idea of what you need where.” CL10 |
|  | - Add ‘Cardiovascular exercise’ as a new page | “...[NuStep] It’s warm-up and cardio, cardio at the beginning of each session…I can’t find any cardio measurement there.” SCI01 |
|  | - Add ‘Reflex integration/nervous system recruitment’ as a new page or component of each activity | “We use reflex integration a lot with probably most of our clients and that’s not part of it. Now, I know that might not be part of activity-based therapy, but for us it is. We often use reflex integration and then go into an exercise based off of what we just helped integrate so I’d say that would be missing for us…it’s teaching their nervous system how to elicit a movement more than muscle strengthening.” CL09 |
|  | - Remove ‘Measures of exertion’ from all activities | “The Borg rating of perceived exertion, I don’t know if I would really ever track my exertion.” SCI05 |
|  | - Keep ‘Measures of exertion’ for only some activities (i.e., loadbearing exercise in standing and overground walking) | “Even when I was able-bodied, I don’t think I’ve ever taken it after every set or rep, but again that’s just my thing. I do know sometimes like even if I do work out and I had a really good set and I feel my blood pressure going through the roof, I might just be like, “Hey, you know do you mind taking my blood pressure or my heart rate?” just to see what I’m getting then. I think the heart rate is very important, but again it would be different if I was actually wearing an Apple watch or a chest monitor or something like that that would record it…For me truthfully, it would be standing stuff just because my blood pressure does go through the roof. When I’m doing strength stuff and whenever I’m on a bench or chair and I’m somewhat grounded, I don’t think it’s as relevant. But I do know at the same time, you need to get your heart rate up in order to get some type of fitness out of your workouts. So, I can see it more for cardio and stuff like that, ‘cause if you’re not getting your heart rate up then you’re essentially not really doing anything for your cardiovascular.” SCI03 |
| Treadmill Training | - Add ‘Walking speed’ for each bout | “I liked that walking speed was in a range or max, but for each bout or rep [it] would be good to have a record for that…each speed for each one.” CL05 |
|  | - Add ‘Level of Difficulty’ for each bout | “We did about a 35-minute stint and he did some forward walking with going and then resting, going and resting, and then he did backwards going, resting, going, resting, and then he did sideways, both ways. So, I felt like I could capture it, but it was hard to capture what he did each direction because it varied for each one…when he’s going forward, he’s at a different speed than when he’s going backwards or sideways… it does change throughout as well… some other clients who get really fatigued through it, I might decrease that speed for part of the session. So, maybe if that was part of the table instead of just an overall piece.” CL09 |
| Overground Walking | - Add ‘Walking distance’ for each bout | “By set, ‘cause that's usually what I look at is how long someone can walk before they require a break.” CL05 |
|  | - Delete unit of measure for ‘Duration of activity’ | “Just a little bit more open-ended for the duration of activity…‘cause it might vary a lot…the unit of time is already put in for minutes. If you leave the unit of time open so that they can put in whatever unit of time they’re using…seconds or hours.” CL08 |
|  | - Add other units of measure for ‘Walking speed’ | “The m/s worked for me, that was what was applicable to my situation. I guess including the km/hour if they’re more.’ CL08 |
|  | - Add ‘Number of steps’ for ‘Walking distance’ | “Walking on the ground doesn’t have any speed…our speed is slow…I think the number of steps is better.” SCI01 |
| Muscle Strengthening | - Add option ‘Whole body’ for ‘Muscles targeted’ | “I did air squats holding a kettle bell on a Bosu ball or on the back of a Bosu ball. So, it becomes a lot… I'm strengthening the upper body, the lower body and the balance. So, when muscles are targeted where it's kind of a whole-body exercise…as for listing all the muscles it can get a little complicated.” CL05 |
|  | - Add FES parameters for each muscle group | “When you do something…complex along with the Xcite with FES, they’re doing multiple muscle groups at one time so I was just wondering if maybe we should have a chart in there with that stuff so that you can capture more, because the frequency or waveform…pulse duration, it might be different for each muscle group that’s working at that time.” CL09 |
|  | - Add ‘Hold duration’ for each bout | “The one thing I could see lacking is just also adding like a hold or a duration if the exercise you’re doing is isometric instead of number of repetitions.” CL08 |
|  | - Add ‘Other’ for external load and ‘Therapist resistance’ | “The only other thing I could say is the resistance bands, I know there’s different colours that mean different tension, but I don’t know how you would actually put that in to something ‘cause different companies have different colours for resistance bands, but there are quite a bit of things that I do use that require resistance bands.” SCI03 |
|  | - Add ‘Position’ and different surface options | “Even if you had a checkmark there or a box, because I actually do a lot of my stuff in a standing frame and it’s muscle-building…So maybe you could just have ‘Are you standing? Sitting? what are you in? are you on a flat bench? are you on the ground?’ Because sometimes I do some stuff off the ledge as well. I do things off the BOSU ball. So there’s multiple ways where muscle strengthening can be done as well.” SCI03 |
|  | - Add option to track ‘Palpable contraction/participation’ or ‘Initiation of spasm/tone’ | “Palpable contraction or palpable participation in the targeted muscles or above the targeted muscles or below the targeted muscle. I’d say like initiation of spasm or tone with participation.” CL03 |
| Ergometer Training | - Add ‘Standing and Kneeling positions’ | “I do a lot of my arm ergometer stuff standing...maybe there should be a box or something where it would be. ‘Cause when I do things seated compared to standing is quite drastically different in the outputs of my exercise, like heart rate, blood pressure…longevity, all that stuff actually is quite a bit different from seated to standing.” SCI03 |
|  | - Add ‘Cadence’ for each bout | “P: This might be in the duration of cycles…like we might do a ladder so it’s 30 seconds on, 30 seconds off, or your intensity goes up every 30 seconds or a minute.  I: So maybe in that top box along with like the number of sets, the rest break and how long you’re doing it for, we should have the intensity as another box where……you can record the speed or the revolutions per minute…if it’s gonna change back and forth.  P: I think that’s good, yeah…some pieces of equipment are just based on levels so it’s not really a power output or watts, it’s just level one is this, level two is that.” SCI03 |
|  | - Add ‘Number of revolutions’ for each bout | “That's how I track it. Yeah, I usually count it as repetitions…if I'm doing spin bike, I use that as we're gonna do sets and reps of rotations.” CL04 |
|  | - Add options for resting position (e.g., in sitting or standing) | “We do the ergometer mostly in a standing frame, so I don’t know if you guys want to [know] like the resting while still standing in the standing frame or if you wanted rest time while he was down in the frame. So, I don’t know if that’s something that you guys would’ve been more interested to learn about or find out about.” CL06 |
| Loadbearing – Standing | - Add ‘Type of surface’ | “Standing on a Bosu, maybe using a TRX as the upper body support, but can stand on a Bosu upside down without any upper body support…the floor how stable or unstable is it might be something you want to record as well.” CL04 |
|  | - Add ‘Level of Difficulty’ for each bout (e.g., none/single/double arm, muscle strengthening, ball throw, weight shifting, mini squats, knee bends, single leg, planking, balance, reaching) | “We have the kind of table at the top with the bout, the minutes and then the rest. You might be able to tack on like another column there saying like what exercises were you doing, if you’re using exercises during those minutes. So sometimes what I'll do is like if we have load bearing minutes for let's say twenty-five I could write in weight shifting or single line support, snow angel or something like that to kind of indicate what was done with the background… additional core exercises or upper extremity exercises…mini squats or sit to stand or knee bends, hip circles, you can do double arms or hip free standing, planking.“ CL03 |
|  | - Add ‘Vibration’ and parameters | “Length of time and then ours is high or low and then the frequency.” CL09 |
| Loadbearing – 4-Point | Add ‘Level of Difficulty’ for each bout (e.g., perturbation, ball tap, cat and dog, balance, push-ups, muscle strengthening, 3-point, burpee) | Maybe you could put in…hip perturbation, so like a section for something like that…we've done it where we set up say three or four balls and then you have to…tap the balls and they're all in different, like one’s to your left, one’s centre, one's a little forward, one’s to your right, one's a little back and things like that…so you have to hold yourself steady while also doing that dynamic movement of the arm…I guess the main thing we do, sometimes like a row while we're in four point. So having it with a resistance band, so the trainer will pull it and I’ll have to try and pull it towards me while staying steady...I guess like pushups also is kinda done in four point.” SCI02 |
| Loadbearing – High kneeling | - Add position ‘low kneel (i.e., hero pose) and high kneel’ for each bout | “Sometimes, we do hero pose and high kneeling. So like with the client I was using the tool with, I did that core routine from a hero pose with him…then we moved into some high kneeling things and we kind of switched back and forth between a high kneeling exercise and a hero pose exercise…So we don't know if there's a different way that you want to note that…you could even just call this page “kneeling” and then have a tick box in each of the bout as is it hero pose or is it high kneeling? And then the trainer can just tick off which one that exercise was in just to be clear.” CL03 |
|  | - Add ‘Level of difficulty’ for each bout (e.g., muscle strengthening, ball throw/hit, single arm, balancing, perturbations, reaching) | “Some clients, we’re practicing just high kneeling and balancing, but with others they can maintain it for a longer period of time so we can add in activities like reaching, or upper extremity weights to challenge their core, or playing catch.” CL01 |
|  | - Add ‘Support overhead’ and ‘Unstable/Stable surface’ | “I've gotten clients to high kneel to a stability ball. Where it's extremely unstable, but it's in front of them. Or anything else, a stability ball, a wheelie bar, that’s also very unstable. So, the level of sturdiness…the support surfaces [of the] upper body to get into high kneel is maybe something that shall also be considered in that section.” CL04 |
| Loadbearing – Crawling | - Add ‘Other’ for distance unit of measure for each bout | “I wouldn’t mark down total distance. I’d just mark down two lengths of mat instead of meters or feet.” SCI04 |
|  | - Add ‘Harness’ to body weight support | “We did high kneeling, but crawling while in high kneeling…we're in a harness belt, so that's helping with like, you could easily put body weight supported, but then I also have to focus on keeping upright and then crawling.” SCI02 |
| Transfer training | - Add ‘Ending seat height’ and ‘Other’ for seat height | “P: So, for transfer training, I know we don't actually measure the height. What we go off of…is like how many box height or mat height, we've gone on up type thing.  I: Okay. So maybe a little comment section where you can add in if it's something other than the units of height measures.  P: Yeah.” SCI02 |
|  | - Add ‘Type of transfer’ as a parameter (e.g., stand and pivot, sit /stand, sit/sit, hero/high kneel, plank/4-point) | “For transfer training, I was wondering if you wanted to add in different types of transfers? So, for example, like some of our clients are working towards the stand and pivot transfer.” CL03 |
|  | - Add ‘Level of difficulty’ (e.g., hard or soft surface) | “I guess it would just be a challenge section on that for whether it's a hard or soft surface.” CL05 |
| Balance Training | - Add ‘Static or Dynamic’ options | “We sometimes categorize between static or dynamic balance. So, I thought those might be two variables you’d be interested in adding in just to determine is the client in a static position or are they working on balancing as well as moving.” CL03 |
|  | - Split ‘Sitting’ into ‘Short and Long sitting’ | “Whether it's short sitting versus long sitting is a very different balance check.” CL05 |
|  | - Add ‘Level of difficulty’ for each bout (e.g., internal/external perturbation, ball throw/catch, one leg) | “The only thing would be to add in if they were balancing on one leg versus two…What else are they doing while they’re working on their balance?... Are they throwing a ball and playing catch or are they lifting a dowel over their head or from side to side?…I would say if you’re looking to get a full picture, putting it into the table would be better.” CL09 |
| Other Task- specific Movements | - Add ‘Duration’ for each bout | “…maybe with the number of reps…maybe reps slash time possibly…So, that can kind of keep you in the general field where you can use this page for everything.” CL10 |
|  | - Add ‘Intensity’ for each bout with unit of measure open | “P: We have a Vita Glider... there’s not a pound per se, it’s level 1, 2, 3, 4, it goes up to 7...It’s just its own…measurement or resistance, but for me to say that it was a 5-lb, 10-lb, I don’t know.  I: …we can add another column beside rest where it has that intensity and then depending on whatever the machine is and what their unit of measure is, you can just put that in.  P: Right.” SCI03 |
